# *Cryptococcus*–Epithelial Interactions

**DOI:** 10.3390/jof3040053

**Published:** 2017-10-02

**Authors:** Leanne M. Taylor-Smith

**Affiliations:** Institute of Microbiology and Infection, School of Biosciences, University of Birmingham, Edgbaston, Birmingham B15 2TT, West Midlands, UK; l.m.taylorsmith@bham.ac.uk

**Keywords:** *Cryptococcus*, epithelial cells, host–fungal interactions

## Abstract

The fungal pathogen, *Cryptococcus neoformans*, causes devastating levels of morbidity and mortality. Infections with this fungus tend to be predominantly in immunocompromised individuals, such as those with HIV. Infections initiate with inhalation of cryptococcal cells and entry of the pathogen into the lungs. The bronchial epithelial cells of the upper airway and the alveolar epithelial cells of the lower airway are likely to be the first host cells that *Cryptococcus* engage with. Thus the interaction of cryptococci and the respiratory epithelia will be the focus of this review. *C. neoformans* has been shown to adhere to respiratory epithelial cells, although if the role of the capsule is in aiding or hindering this adhesion is debatable. The epithelia are also able to react to cryptococci with the release of cytokines and chemokines to start the immune response to this invading pathogen. The activity of surfactant components that line this mucosal barrier towards *Cryptococcus* and the metabolic and transcriptional reaction of cryptococci when encountering epithelial cells will also be discussed.

## 1. Introduction

*Cryptococcus neoformans* is a basidiomycete fungus capable of causing fatal infections in humans. It is estimated that *C. neoformans* causes over 200,000 infections per year with the majority of these occurring in sub-Saharan Africa where HIV rates are high [1,2,3]. *C. neoformans* is most commonly known to infect individuals who are immunocompromised, however cases of infections in immunocompetent patients do exist [4]. Infections are thought to initiate with inhalation of infectious particles likely to be spores or desiccated yeasts. For many a short and often asymptomatic pulmonary infection will be as serious as this infection gets. However for immunocompromised individuals infection can continue to disseminate from the lungs, or perhaps a re-activation of latent cryptococcal cells, leading to often fatal cryptococcosis culminating in cryptococcal meningitis. 

*Cryptococcus neoformans* possesses many virulence factors, but the polysaccharide capsule is considered one of its most vital to causing disease [5,6]. The capsule is composed mostly (approximately 90%) of glucuronoxylomannan (GXM) with smaller proportions of glucuronoxylomannogalactan (GXMGal) and mannoproteins [5]. The capsule has been shown to be antiphagocytic [7], offer protection against oxidative killing [8] and impair T-cell proliferation [9], to name just a few properties. Exactly how much capsule is present on cryptococcal cells as they enter the body for the first time is debatable but it is known that cryptococci are able to induce capsule production in response to the lung environment [10,11].

**Figure 1:**
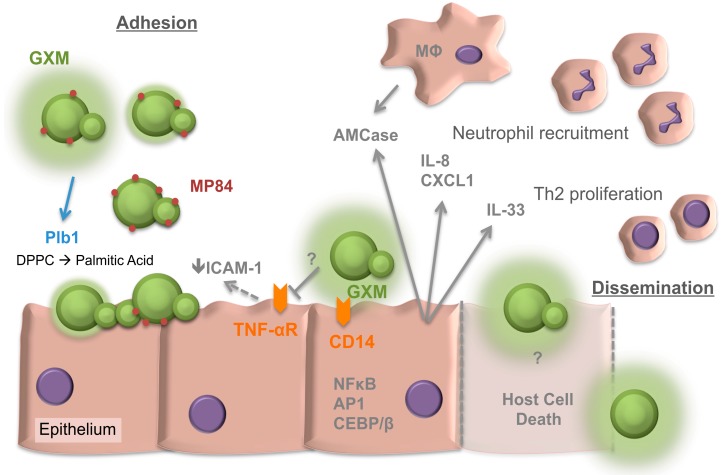
Overview of *Cryptococcus* interactions with the respiratory epithelia. Factors involved in both adhesion and later dissemination are depicted. GXM, glucuronoxylomannan. MP84, Mannoprotein 84. Plb1, phospholipase 1. DPPC, dipalmitoyl phosphatidylcholine. ICAM-1, intercellular adhesion molecule-1. TNF-αR, tumor necrosis factor-alpha receptor. AMCase, acidic mammalian chitinase. IL, interleukin. CD, cluster of differentiation. CXCL1, chemokine (C-X-C motif) ligand 1. Th2, T-helper 2 cell. MΦ, macrophage. NF-κB, nuclear factor kappa-light-chain-enhancer of activated B cells. AP1, Activator protein 1. CEBP/β, CAAT/ enhancer-binding protein β. T arrows, blocking of response. Solid arrows, secretion or inducing action. Dashed arrows, uncertain if activity is direct or indirect. Dashed lines represent host cell death.

Due to its association with HIV infected individuals much attention has been given to the role of the adaptive immunity in cryptococcosis. Innate immunity research has focused mostly on phagocytes, particularly macrophages as they can have such a detrimental effect on infection in mouse models and are therefore believed to be a central determinant of cryptococcal infection [6,12,13]. A rather neglected component of innate immunity that has been rather over looked is the epithelial cells of the respiratory tract. Bronchial epithelial cells of the upper airway and alveolar Type I and Type II epithelial cells form a physical barrier between the outside environment and the tissues beneath them [14,15]. This is not only a physical barrier but also a sensing barrier that has the ability to raise the immunological alarm very early on in infection with the release of an array of cytokines and chemokines (Figure 1). Due to *Cryptococcus* entering the body via the respiratory tract, the airway epithelia are likely to be the first host cells cryptococci have close contact with and are therefore the focus of this review.

## 2. Adherence of *Cryptococcus neoformans* to the Respiratory Epithelia

Much of the work on *Cryptococcus*–epithelial cell interactions has been focused on adhesion. This is of course with good reason as adhesion of microbial cells to host surfaces is critical to the onset of many infections. Most studies have looked to answer two important questions. Firstly, does *C. neoformans* adhere to the epithelial cells of the upper and lower respiratory tract? Secondly, what receptors and ligands are required for this interaction? In the pursuit to answer these questions conflicting and confusing results have been plentiful.

Early investigations indicated lactosylceramide, a glycophospholipid common to epithelial cell membranes, as a possible receptor for the binding of not only *C. neoformans* but also *Candida albicans*, *Histoplasma capsulatum* and *Sporotrichum schenckii* in their yeast forms [16]. However, later findings demonstrated that cryptococcal adherence to rat epithelial cells in culture was likely to be protein based as it could be abolished with trypsin treatment [17]. A large number of papers using the A549 cell line were published from the mid-1990s onwards. This human epithelial type II cell line has paved the way for countless research projects investigating the interactions of microbes and the human epithelia. Initial findings from Merkel and colleagues demonstrated that both encapsulated and acapsular *C. neoformans* could adhere to A549 epithelial cells in a series of radiometric assays [18]. However, an increased adherence was seen with the *cap67* acapsular mutant, a finding also corroborated with a more recent paper [15]. In addition, a temperature sensitive element to the adherence was recorded. Cryptococcal cells grown at 37 °C adhered to epithelial cells greater than those grown at 25 °C, despite 25 °C grown cryptococci producing a smaller capsule. The age of the culture also affected adherence, with older cultures adhering more to A549 cells. Altered adherence was also seen when *C. neoformans* was grown with different carbon sources, but with variability across different strains [18]. The results overall indicate a cryptococcal ligand that is not just unmasked with reduced capsule size but is also only present in a time and temperature dependent manner.

Almost 10 years later Barbosa et al., used the A549 cell line once more to investigate cryptococcal adhesion to epithelial cells [19]. The authors demonstrated that the adhesion of *Cryptococcus* to A549 cells could be blocked with the use of an antibody. This is a now well-known antibody to the *Cryptococcus* field, the mouse IgG1 18B7 antibody raised against cryptococcal GXM by the Casadevall lab [20]. Pre-treating either the cryptococcal yeasts or the epithelial cells before co-incubation resulted in reduced adhesion between the two, indicating GXM as a crucial ligand [19]. The group also show that GXM is able to bind the epithelial cells directly, further supporting a conclusion that epithelial cells can recognise GXM. These results however do seem to oppose those of Merkel et al., who showed that a lack of capsule (mostly made of GXM) resulted in increased adhesion of *Cryptococcus* [18]. Possible explanations could be the different strains of *C. neoformans* used but also the growth conditions of the cryptococci before presenting to epithelial cells. But this also poses an important question; what growth conditions of cryptococci provide us with cryptococcal cells in a form most similar to that which is inhaled at the start of infection?

Another candidate adhesion factor was also presented in 2006 by Ganendren and co-workers. Phopholipase B (PLB1) is a secreted cryptococcal enzyme with three enzymatic activities; phospholipase, lysophospholipase and lysophospholipase transacylase [21]. The parental wild type H99, mutant Δ*plb1* and reconstituted Δ*plb1^rec^* were all co-incubated with A549 cells to see the importance of PLB1 in binding to epithelial cells. It was found that Δ*plb1* had a reduced adherence to A549 cells when compared to H99 and Δ*plb1^rec^* [22]. Using chemical inhibitors of each of the three enzymatic activities of Plb1, only blocking the phospholipase activity was found to reduce the adhesion of H99 and the reconstituted Δ*plb1^rec^*. The role of Plb1 in adhesion could be due to phospholipase providing fatty acids. The authors postulate that the in vivo mechanism could be Plb1 phospholipase activity on dipalmitoyl phosphatidylcholine (DPPC), a major component of lung surfactant, to create glycerophosphocholine and palmitic acid. This idea is supported by the dose dependent increase in adhesion when palmitic acid is added to the system with the Δ*plb1* mutant [22]. Further supporting the importance of Plb1 as a virulence factor in the lung, Plb1 has been determined as an important factor for the dissemination of cryptococci from the lungs of mice [23].

One of the more recent publications on epithelial cell adhesion perhaps sheds light on the previous conflicting results on the role of capsule GXM. It was demonstrated that wild type capsule producing strains use GXM to aid adherence, however, the acapsular mutants are more reliant on mannoprotein 84 (MP84) for adhesion to A549 cells [24]. Although there is still some confusion in the field, it would seem most likely that a combination of all these ligands play a role in adhesion of cryptococci to epithelial cells but the most dominant components are determined by the age, capsule size, growth stage and strain of the *Cryptococcus* involved.

## 3. Internalisation of Cryptococci by the Epithelia

After adherence to host membranes the next step for an invasive infection is dissemination across that barrier. There are multiple ways in which *C. neoformans* could leave the lungs. One theory is that cryptococci hijack a ride within alveolar macrophages [10]. An alternative is the translocation of cryptococci across the alveolar epithelia. Although this phenomenon has not been studied in great detail, the internalisation of cryptococci into epithelial cells has been recorded by many groups [15,18,19,22,25]. With a mixture of techniques including electron microscopy, confocal microscopy and flow cytometry, it appears that epithelial cell internalisation of cryptococci is a rare event and the relevance of this to infection is currently unknown. An interaction perhaps more likely to play a significant role in dissemination from the lungs is the disruption of the epithelial barrier by induced epithelial cell death. Barbosa and colleagues not only recorded some internalisation of *C. neoformans* into A549 cells but they also suspect that these events resulted in host cell death. In addition, the group demonstrated that incubating cryptococci with epithelial cells for prolonged periods of time resulted in significant levels of host cell death, as measured by lactate dehydrogenase assay [19]. Such cell death events is likely to explain the lung lesions seen by others in the mouse model for respiratory infection [23]. The fungal component responsible for the cell death is as yet undetermined, but the authors were able to demonstrate it is unlikely to be the capsule component GXM as purified GXM alone was unable to mimic the response [19].

## 4. The Response of the Respiratory Epithelia to *Cryptococcus*

As mentioned previously, the respiratory epithelia has the opportunity to detect and respond to invading pathogens with the production of cytokines and chemokines. The major component of the cryptococcal capsule, GXM, also appears to have a role in inducing cytokine production from epithelial cells. It was demonstrated that GXM is able to bind CD14 on epithelial cells which in turn induces IL-8 release [26]. The chemokine IL-8 is typically associated with neutrophil recruitment and likely crucial for the immune response and clearance of infections. This is actually a rare case of *C. neoformans* eliciting a pro-inflammatory response, as it is so well associated with successfully dampening the response and remaining under the immunological radar.

Research by Guillot et al., used the BEAS-2B bronchial epithelial cell line to more closely mimic the epithelia of the upper respiratory tract, cells that could come into contact with *Cryptococcus* even earlier than the type I and type II epithelia of the alveolae. These bronchial epithelial cells were also able to produce IL-8 in response to *C. neoformans*, however only when using the acapsular mutant grown at 37 °C [25]. This IL-8 secretion was AP-1 and NF-κB dependent. It was also found that bronchial cells released the CXCL1 chemokine and upregulated the expression of the CAAT/enhancer-binding protein β (CEBP/β) transcription factor [25]. CEBP/β may be binding co-operatively with NF-κB to activate IL-8 gene expression [27]. Interestingly the group were also able to study the responses of primary normal human bronchial epithelial cells (NHBE). These cells responded well, producing the more IL-8 than the BEAS-2B cells but also exhibiting more cell damage. More curiously, the primary cells were able to produce IL-8 to similar levels in response to both encapsulated and acapsular *C. neoformans.* Although, the most cell death was seen with acapsular *Cryptococcus*. The primary bronchial cells responded similarly to both forms of cryptococci in terms of CXCL1 production [25]. The authors propose the lack of response from BEAS-2B cells to encapsulated strains is likely to be because of the capsule and also the lack of physical contact and adherence. This is supported by some previous work already discussed in this review that demonstrates acapsular strains binding far more readily to epithelial cells [15,18]. However it would not agree with some work showing GXM as a crucial component to not only adherence but also specifically IL-8 release [19,24,25,26]. It is possible that the respiratory epithelia comes into contact with both encapsulated and acapsular forms of *Cryptococcus.* In addition, cryptococcal strains differ greatly in capsule size and composition [28,29,30,31]. Capsule is also likely to be induced rapidly upon the change of environment the cryptococci face after inhalation [32]. So either by strain variation or simply the amount of time that has passed since inhalation of infectious propagules, the epithelia will see capsule of various sizes. How quickly a non-capsulated *Cryptococcus* is able to respond to the host environment and produce a thick capsule may have huge consequences to the progression of infection.

Other work investigating the epithelial response by Piehler et al., and Flaczyk et al., has shown that IL-33 is produced during mouse lung infections with *C. neoformans* [33,34,35]. The IL-33 receptor T1/ST2 is also up-regulated on T-helper 2 (Th2) cells [33]. The resulting signalling from IL-33:T1/ST2 engagement is likely to contribute to dissemination of cryptococci from the lung [33,35]. Th2 responses have long been associated with survival and dissemination of cryptococci [33,36]. IL-33 is seen as a propagator of Th2 immunity by activation of ILC2s (innate lymphoid cells) that produce IL-5 and IL-13, thus inducing proliferation of Th2 cells [34]. However the source of this IL-33 remained unknown until recently. Heyen and co-workers show that during *C. neoformans* infection of the mouse lung, the alveolar type II epithelial cells are the greatest source of IL-33 [37]. The IL-33 also resulted in down regulation of E-cadherin on epithelial cells, required for maintaining epithelial barrier integrity. It is not yet elucidated if the loss of E-cadherin was orchestrated by IL-33 directly or indirectly. If indirect this could be via IL-4 and IL-13 [37]. The permeability of the epithelial barrier due to loss of E-cadherin was not specifically tested. Although it is interesting to speculate, as did the authors, if barrier integrity is lost would this allow dissemination of *Cryptococcus* or more immune cell infiltration to aid clearing? Further results from this study showed that cryptococcal exposed pulmonary epithelial cells also had increased mRNA for the surfactant protein C (SP-C) and CXCL15 (Mouse neutrophil chemokine), but this was independent of IL-33. The authors hypothesise that SP-C may have role in increasing the phagocytosis of *Cryptococcus*, via SP–C: CD14 interactions [38]. CD14 is also known to recognise GXM, as previously discussed [26], therefore SP-C may increase binding of CD14 to GXM. 

The influence of cryptococci on epithelial cell ligand expression and how this might influence immune cell recruitment has also been investigated. Intercellular adhesion molecule-1 (ICAM-1) expression on epithelial cells can be increased via TNFα [39]. ICAM-1 can help the attachment of monocytes expressing CR3 ligand, or neutrophils, B cells and T-cells that express LFA-1 ligand, helping recruitment and perhaps keeping cells there until local infection is cleared. Lowering ICAM-1 expression might affect phagocyte migration but also antigen presentation to T and B cells. Rather than looking at direct adherence of cryptococci to epithelial cells this time Merkel et al., investigated the effect of cryptococcal secreted products on epithelial cells. It was discovered that the heat stable secreted products of *C. neoformans* were able to interfere with TNF-α orchestrated ICAM-1 expression [39]. The authors propose that this is not cryptococcal components inducing ICAM-1 shedding, but is more likely to be due to blocking the TNF-α receptor or causing loss of the TNF-α receptor.

A slightly different approach was taken by Goldman et al., who explored the idea that illnesses such as asthma and allergic respiratory syndromes could be more likely to occur in individuals with prior exposure to a fungal pathogen [40]. Most immunocompetent people are likely to have had a nonsymptomatic infection of *C. neoformans* [41]. It also follows that Th2 responses have been linked to allergic respiratory illnesses, and *C. neoformans* is known to promote a Th2 biased immune reaction [36,42]. Indeed, cryptococcal infection of the rat lung was shown to enhance the allergic response to ovalbumin as measured by ovalbumin specific IgE production in serum and eosinophil counts in bronchoalveolar lavage [40]. This work also looked at the various stages of infection, comparing cleared, local persistent and disseminated systemic infections. Short-term (2 weeks) cryptococcal infections resulted in increased production of IL-10, TNF-α and IL-13 in the lungs, a mixture of both pro and anti-inflammatory cytokines. On the other hand, systemic infections had low IL-13 and high IFNγ in the lungs [40]. Interestingly, the acapsular mutant strain was unable to induce high IgE levels, but produced the same cytokine signature as wild type. The source of the cytokines within the lung were not determined in this particular study.

The role of host chitinases has also been explored. Using a chronic pulmonary infection model in rats it was found that *C. neoformans* was able to induce a two fold increase in the chitinase activity of the bronchoalveolar lavage fluid by two days post-infection. This chitinase activity continued to rise during infection and peaked at six fold at fourteen days post infection. It is thought that most of the chitinase activity is due to host acidic mammalian chitinase (AMCase) rather than other host chitinases [43]. The source of AMCase would likely be from both alveolar epithelial cells and macrophages. Much of the research so far has focussed on determining the immune response of host cells when they encounter *Cryptococcus*, which is of course very important to know, however we should also look for potential immune modulation techniques to aid host cells in the rapid clearance of cryptococci. This approach is however complicated by the fact that many cryptococcal infections are in immunocompromised individuals that may require alternative approaches to those required in otherwise healthy individuals. Such immunomodulation techniques could have potential therapeutic value against not only *Cryptococcus* infections but also other fungal pathogens that elicit a similar immune reaction from the host.

## 5. The Cryptococcal Response to the Respiratory Niche

Much of this review so far has focused on the host response to *Cryptococcus* lung infection, but this section will instead discuss the response of the cryptococcal cells themselves. EM analysis of mouse lungs at various time points post infection with *C. neoformans* showed that both cell wall and cell body size increased over the course of a 28 day infection [32]. The cell wall increase may be due to melanisation during infection, as the cryptococci were seen to get progressively darker in microscopy of lung sections. Furthermore, giant cells up to a maximum of 28 µm in diameter were seen—one of the first descriptions (along with some clinical case reports of the 70s and 80s [44]) of what we now know as cryptococcal Titan cells [11,45,46]. 

Cryptococcal Titan cells are enlarged cells of cryptococci measuring up to 100 µm in diameter [11,45,47]. This response of cryptococci to the in vivo conditions found within the lungs is thought to be a general virulence mechanism for survival. Not only are Titan cells more virulent in mouse models [46], but are also more resistant to many environmental stresses. The large size of Titan cells makes phagocytosis of these cells physically impossible [45]. Moreover, Titan cells are polyploidy and retain the ability to form normal size daughter cells, thought to provide a genetic ‘mixing pot’ to allow for the survival of new and improved daughter cells in stress conditions [11]. This is a reasonably new area of *Cryptococcus* research and it will be interesting to see future progress in determining the factors responsible for initiation of Titan cell morphology and the overall effect of Titan cells on the course of infections in humans.

To investigate the metabolic response of cryptococci Hu et al., employed Serial analysis of gene expression (SAGE) to analyse *C. neoformans* rescued from murine lung infections at 8 and 24 h after infection initiation. Most striking was the increased expression of genes involved in carbon metabolism. More specifically this group of genes included those required for acetyl-CoA metabolism [48]. Further investigations with the *Δacs1* (acetyl-CoA synthase) mutant strain determined the strain to be attenuated in virulence, indicating a requirement to utilise acetyl-CoA for full virulence of *Cryptococcus* in pulmonary infections. The authors believe the only moderate defect of *Δacs1* is likely due to compensation by other pathways to make acetyl-CoA. It was also hypothesised that the increased biosynthesis of acetyl-CoA could be to allow for chitin and capsule production, as acetyl-CoA is a precursor for chitin and required for O-acetylation of capsule [48].

Can metabolites be identified that are associated with cryptococcal infection of the lung that could have the potential to be used to identify cryptococcal pulmonary infections? This is the question Liew and colleagues set out to answer. The group infected the lung epithelial type II cell line A549 with cryptococci and analysed the supernatants collected over 18 h with Gas chromatography—Mass spectroscopy (GC-MS) [49]. Of note, the metabolites from host cells and *Cryptococcus* cells were not separated, but the authors assume that due to the increased number of cryptococci (multiplicity of infection, MOI, of either 10:1 or 100:1) that the majority of metabolite changes will be due to those produced by the cryptococci. They identified 10 metabolites as important markers of *Cryptococcus*—epithelial cell interactions, which included carbon metabolism and amino acid biosynthesis pathways [49]. Infections with a higher MOI specifically affected β-alanine metabolism and increased the secretion of Pantothenic acid. These are very likely to be from cryptococci as β-alanine and pantothenic acid cannot be secreted from A549 cells. Interestingly, β-alanine biosynthesis is not known to occur in many fungi and is thought to be acquired exogenously. In addition, pantothenic acid production has been previously reported for *Cryptococcus* when grown in low glucose conditions, so it is feasible to conclude these products are truly from the *Cryptococcus* cells and not host cells [50]. It will be intriguing to see if these metabolomics findings could result in a less invasive or faster method for identification of *Cryptococcus* infections.

## 6. Collectins of the Respiratory Lining and *Cryptococcus*

Collectins are collagen-containing C-type lectins and can be considered as soluble pattern recognition receptors important to mucosal defences. Upon binding microbes they act to aid the innate immune response in a variety of ways including agglutination, opsonisation, activation of phagocytes or inhibiting microbial growth. The major source of collectins and other surfactant proteins in the lungs is the type II alveolar epithelial cells [51]. Several collectins found within the lung mucosa have been demonstrated to bind *C. neoformans* [52]. The collectins surfactant protein-A (SP-A), surfactant protein-D (SP-D), mannose binding protein and collectin-43 (CL-43) are able to bind acapsular cryptococci in a calcium dependent manner. More specifically, purified SP-D could agglutinate acapsular cryptococci in a dose-dependent manner [52]. Agglutination of microbes could help physical clearance from the airway by mucocillary beating.

Work completed almost a decade later confirmed some of these findings. Again, acapsular cryptococci were able to be bound by SP-D and this could cause agglutination of cryptococcal cells, but this paper also reports some binding of SP-D to encapsulated strains of both serotype A and D [53]. The authors go a step further to determine the mechanism of SP-D binding to cryptococci. GXM and mannose protein 1 were bound with high affinity. Furthermore, binding of SP-D could be inhibited with pre-treatment of *Cryptococcus* with purified GXM. Intact capsule but also shed GXM is therefore likely to bind SP-D, with shed GXM likely to interfere with the immune functions of SP-D [53].

Giles et al., confirm that in vitro SP-A does not bind wild type encapsulated *C. neoformans* H99 [54]. However, come binding was seen if cryptococci were incubated in BAL fluid when exposed to SP-A, suggesting there is something else in lung fluid that mediates the binding. The group performed intranasal infections in WT and SP-A^−/−^ mice but witnessed no significant differences in TNF-α release, fungal load in the lungs or survival outcome for the animals. The authors conclude that SP-A has little relevance in *Cryptococcus* infection [54].

Geunes-Boyer and colleagues were also able to demonstrate SP-D binding to cryptococci. In addition, the team were able to show that SP-D bound the acapsular strain of H99 approximately six times more that the wild type version of *C. neoformans* [55]. But also show that capsule components are a possible ligand for SP-D as they can block SP-D deposition. When the acapsular strain was pre-treated with SP-D it was engulfed more into the J774 murine macrophage cell line than non-treated cryptococci. These internalised acapsular *C. neoformans* were in lysosomal compartments but fewer co-localised with lysosomal-associated membrane protein-1 (LAMP-1). Moreover, when coated in SP-D *C. neoformans* was more able to survive inside macrophages as measured by CFUs. The SP-D^−/−^ mouse was infected intranasally with alexa fluor-488 labelled cryptococci and confocal imaging performed on the tissue sections. Indeed, as seen in vitro, the SP-D positive alveolar macrophages contained far more cryptococci than macrophages in SP-D negative mice [55]. 

These investigations were followed up just a few years later when the same group published their results after looking at the in vivo importance of SP-D in more detail. They found that SP-D^−/−^ mice had delayed time to death when infected with *C. neoformans* and lower fungal loads compared to wild type mice, suggesting a protection by lack of SP-D [56]. Without SP-D there was also a delayed dissemination to the spleen and brain. Normal virulence of cryptococci could be achieved by adding SP-D exogenously and it was shown that SP-D can bind directly to cryptococci provide protection against macrophages and H_2_O_2_ stress. Interestingly, incubating crypto with SP-D in vitro increased the size of the cryptococcal capsule and cryptococci recovered from BAL at 3 days post infection of the SP-D^−/−^ mice seemed to have a ‘leaky capsule’ by India ink staining, suggesting SP-D has the ability to increase capsule size and density [56]. Overall this study provides more evidence that *C. neoformans* can use a host factor whose normal function is to protect the host to its own advantage to better survive within the lung to the ultimate detriment of the host. Early studies showed an opsonic behaviour of SP-D toward cryptococci that would normally be considered beneficial to the host in controlling infection. However, *C. neoformans* is able to exploit the intracellular niche within macrophages [57] and also undergo escape via vomocytosis [58,59], thus this exploitation of SP-D is potentially beneficial for the fungus to enhance proliferation.

## 7. Conclusions

The lung and bronchial epithelium form a physical barrier between the tissue and external environment. These epithelial cells are also actively antimicrobial as part of the innate immune response. Epithelial cells are able to initiate the defence against pathogens with a chemokine and cytokine response. *C. neoformans* is able to adhere to, be engulfed by and induce cell death of epithelial cells. Moreover, cryptococci can modulate changes in epithelial cell ligand expression and secretion of molecules important to the immune response in the lung. The molecular detail underlying many of these responses is not entirely clear at this stage. Some of the conflicting findings regarding the involvement of capsule in many of these processes could be due to many things. Not only is the capsule variable between strains, age of cultures and under various growth parameters but the GXM isolated from these cryptococci can vary structurally depending on how it has been purified [60]. In addition cell wall anchored and shed GXM is likely to be different structurally [60]. Another variation in *Cryptococcus* morphology to consider is the formation of Titan cells. Now we know Titan cells are formed in vivo it would be interesting to look at these cells and their interaction with epithelial cells and phagocytes of the airway as at least a small proportion of cryptococci are likely to be in Titan morphology soon after entering the lungs. Methods to create Titan cells in vitro to enable such studies without the rescue of Titan cells from mice are currently under development by several laboratories. In addition, the role of *Cryptococcus* spores rather than yeast cells in commencing infection in the lung has to date been neglected. An investigation into the varied roles of yeast, Titan and spore cryptococcal cells would be very valuable to the field.

Despite major progress in determining the responses of both cryptococcal yeast and epithelial cells when they interact, the molecular detail behind many of these responses remains to be elucidated. More recent technological advances such as single cell RNA sequencing could help to determine not only the signalling pathways initiated in cells, but also the differential ways specific subtypes of cells are initiated when they encounter *Cryptococcus*. In addition, advances in human cell engineering, such as stem cell technologies and organoid culture, could allow for more detailed single cell level exploration of *Cryptococcus* pathogenesis in the context of human cells.
